# Impact of acute kidney injury in expanded criteria deceased donors on post-transplant clinical outcomes: multicenter cohort study

**DOI:** 10.1186/s12882-019-1225-1

**Published:** 2019-02-04

**Authors:** Woo Yeong Park, Min-Seok Choi, Young Soo Kim, Bum Soon Choi, Cheol Whee Park, Chul Woo Yang, Yong-Soo Kim, Kyubok Jin, Seungyeup Han, Byung Ha Chung

**Affiliations:** 1 Transplant Research Center, Seoul, South Korea; 2 Division of Nephrology, Department of Internal Medicine, Seoul St. Mary’s Hospital, College of Medicine, The Catholic University of Korea, Seoul, South Korea; 3 Division of Nephrology, Department of Internal Medicine, Uijeongbu St. Mary’s hospital, College of Medicine, The Catholic University of Korea, Uijeongbu, South Korea; 4 Department of Internal Medicine, Keimyung University School of Medicine, Daegu, South Korea; 5 Keimyung University Kidney Institute, Daegu, South Korea

**Keywords:** Expanded criteria donor, Acute kidney injury, Deceased donor, Kidney transplantation

## Abstract

**Background:**

The problem of organ shortage is an important issue in kidney transplantation, but the effect of kidney donation on AKI is unclear. The aim of this study was to investigate the impact of acute kidney injury (AKI) on post-transplant clinical outcomes for deceased donor kidney transplantation (DDKT) using standard criteria donors (SCDs) versus expanded criteria donors (ECDs).

**Methods:**

Five-hundred nine KT recipients receiving kidneys from 386 deceased donors (DDs) were included from three transplant centers. Recipients were classified into the SCD-KT or ECD-KT group according to corresponding DDs and both groups were divided into the AKI-KT or non-AKI-KT subgroups according to AKI in donor. We compared the clinical outcomes among those four groups and investigated the interaction between AKI in donors and ECD on allograft outcome.

**Results:**

The incidence of delayed allograft function was higher when the donors had AKI within SCD-KT and ECD-KT groups. In allograft biopsies within 3 months, chronic change was more significant in the AKI-ECD-KT subgroup than in the non-AKI-ECD-KT subgroup, but it did not differ between AKI-SCD-KT and non-AKI-SCD-KT group. AKI-ECD-KT showed higher risk for death-censored allograft failure than the other three groups and a significant interaction was observed between AKI in donors and ECD on the allograft outcome.

**Conclusions:**

The presence of AKI in ECDs significantly impacted the long-term allograft outcomes of kidney transplant recipients, but it did not in SCDs.

**Electronic supplementary material:**

The online version of this article (10.1186/s12882-019-1225-1) contains supplementary material, which is available to authorized users.

## Background

The imbalance between donors and recipients of kidney transplantation (KT) needs the increase of the potential donor pool for transplantation [1–7]. In this regard, the use of kidneys from expanded criteria donors (ECDs) in deceased donor (DD) KT has been proposed as an important strategy for solving this donor shortage [7–9]. Previous studies have demonstrated that the prognosis of KT from ECDs is not significantly different from that of KT from standard criteria donors (SCDs) [10, 11]. In contrast, other studies have reported that KT from ECDs yielded poorer clinical outcomes in terms of allograft survival rate compared with that from SCDs [12, 13]. Therefore, it is unclear whether DDKT from ECDs shows comparable clinical outcomes to that from SCDs.

Acute kidney injury (AKI) is very common in recently deceased individuals [14, 15]. We and others have shown that, although AKI in DDs results in higher incidence of delayed graft function (DGF) in their corresponding recipients, it does not significantly impact long-term allograft survival [16, 17]. In contrast, other studies showed that AKI does impact long-term allograft outcomes [18]. Therefore, it is also unclear whether AKI itself has a significant impact on allograft outcomes.

With respect to AKI in the normal context, long-term renal outcomes largely depend on the underlying status of the kidney; therefore, the renal outcomes of AKI in underlying chronic kidney disease (CKD) are significantly worse than those of AKI in normal kidneys [19, 20]. Therefore, it is possible that AKI in ECDs, who might have underlying CKD, has a more significant impact on long-term allograft survival than AKI in SCDs, who might have less severe or no underlying CKD.

Based on these findings, we aimed to investigate whether the impact of AKI in DDs on the post-transplant clinical outcomes differs between KT from SCDs (SCD-KT) and those from ECDs (ECD-KT). To this end, we compared clinical outcomes including the incidence of DGF, allograft survival, and patient survival for KT from donors with AKI versus those for KT from non-AKI donors between the SCD-KT and ECD-KT groups, and also investigated the interaction between AKI in donors and ECD on allograft survival.

## Methods

### Study population

Five-hundred and nine kidney transplant recipients (KTRs) receiving kidneys from 386 DDs were included. The KTRs were treated at three transplant centers (A, Seoul St. Mary’s Hospital, B, Uijeongbu St. Mary’s Hospital, and C, Keimyung University Hospital) between October 1996 and May 2016. ECD was defined according to the United Network for Organ Sharing (UNOS) criteria [21]. AKI was diagnosed according to the Kidney Disease: Improving Global Outcomes (KDIGO) criteria. This criteria defines that AKI is an increase in a serum creatinine (SCr) level by ≥0.3 mg/dL (26.5 μmol/L) within 48 h or an increase in a SCr level to ≥1.5 times baseline that had already been known or presumed to have developed within 7 days, or a reduction in urine output (< 0.5 mL/kg/hour for 6 h). The stages of AKI were defined according to the severity of an increase in a SCr level or a reduction in urine output [17, 22].

### Clinical parameters and outcomes

We retrospectively analyzed the donor and recipient data. The donor data included age, sex, body mass index (BMI) (kg/m^2^), history of diabetes mellitus (DM) and hypertension (HTN), cause of death, and estimated glomerular filtration rate (eGFR). The recipient data included age, sex, BMI, history and duration of dialysis before KT, number of previous KTs, cause of end-stage renal disease, history of DM and HTN, cold ischemic time, number of human leukocyte antigen (HLA) mismatches, immunosuppressant type and percentage of panel-reactive antibodies (PRAs). We also analyzed the findings of allograft biopsies obtained within 3 months from KT in KTRs, and analyzed the extent of chronic damage (interstitial fibrosis (IF, ci > 0), tubular atrophy (TA, ct > 0), glomerulosclerosis (GS)) in the allograft tissue. We calculated the average score of IF and TA in each group [23]. DGF was defined as dialysis requirement within the first week after KT [24].

The primary outcome was to investigate the impact of AKI on the death-censored allograft survival for DDKT using SCDs versus ECDs, and analyze the interaction between AKI in DDs and ECDs. The secondary outcomes were to investigate the incidence of DGF, the change of allograft function by eGFR calculated using the modification of diet in renal disease (MDRD) equation until 1 year after KT [25], the chronic change in allograft tissue by allograft kidney biopsies performed within 3 months after KT, and the patient survival between SCD-KT and ECD-KT and between the AKI-KT and non-AKI-KT subgroups, respectively.

This study was approved by the institutional review boards of Seoul St. Mary’s Hospital (XC15RIMI0061K), Uijeongbu St. Mary’s Hospital (XC15RIMI0061U), and Keimyung University School of Medicine, Dongsan Medical Center (2017–08-019).

### Statistical analysis

Student’s *t*-test was used for the analysis of continuous variables with a normal distribution, and those were expressed as the mean ± standard deviation. On the contrary, the Mann-Whitney test was used for the analysis of those with a non-normal distribution. The Chi-square test or Fisher’s exact test was used to analyze categorical variables, and those were expressed as the number and percentage. Kaplan-Meier curves and log-rank tests were used for the description of the death-censored graft survival and patient survival. Logistic regression analysis was used to investigate whether AKI in DDs or ECD is an independent risk factor for the development of DGF and recipient age, transplant year (1996~2005 vs. 2006~2010 vs. 2011~2016), transplant center, prior KT, recipient diabetes, HLA mismatch number, high PRA, donor gender were included as confounding variables. Cox proportional hazards regression analysis was used to investigate whether AKI in ECD is an independent risk factor for death censored allograft failure. Interaction effects between donor type (ECD vs. SCD) and AKI in donors were explored by adding interaction terms to the model in which donor type was treated as an ordinal in overall population. *P*-values < 0.05 were considered statistically significant. All statistical analyses were performed using SPSS 19.0 software (SPSS Inc., Chicago, IL, USA) and the statistical package MedCalc version 15.5 (MedCalc, Mariakerke, Belgium).

## Results

**Fig. 1:**
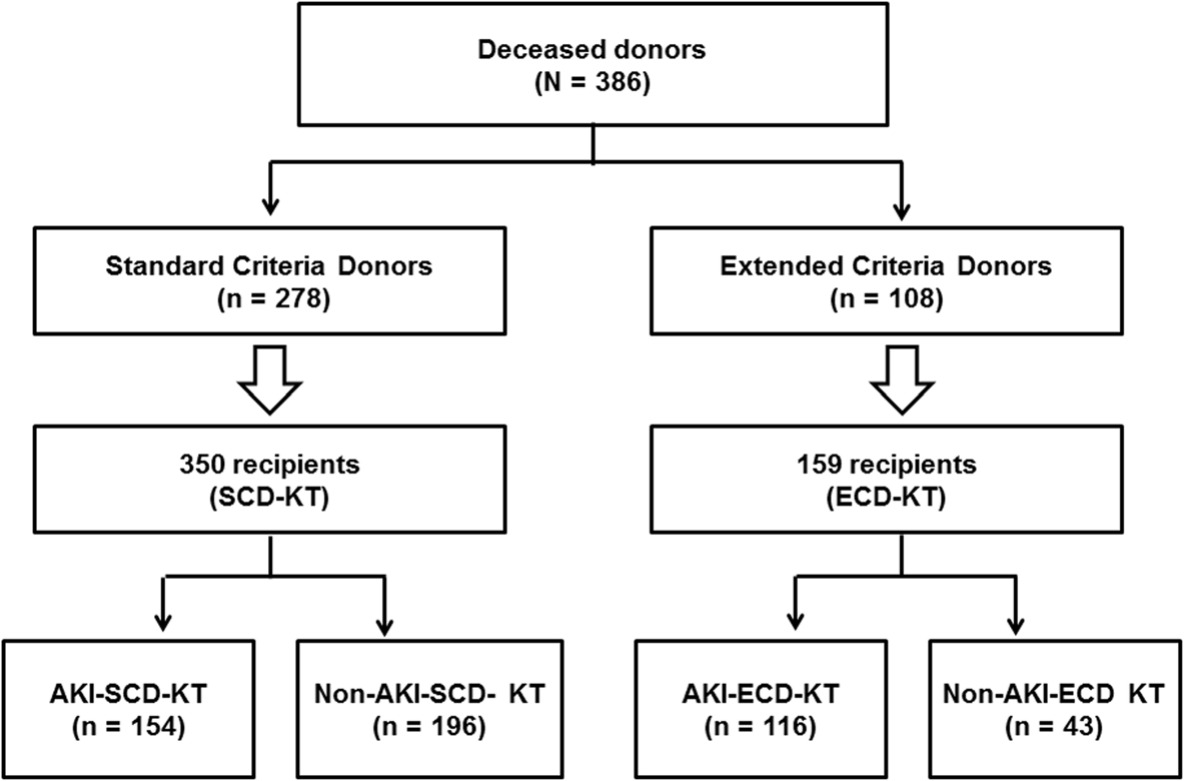
Patient algorithm and distribution in this study. KTRs were classified into the SCD-KT group or the ECD-KT group according to the status of their corresponding donors. In addition, each group was subdivided into an AKI subgroup and a non-AKI subgroup according to the presence of AKI (as assessed by the KDIGO criteria) in the corresponding DDs. Abbreviations: KTRs, kidney transplant recipients; SCD, standard criteria donor; ECD, expanded criteria donor; KT, kidney transplantation; AKI, acute kidney injury; KDIGO, kidney disease: improving global outcome; DDs, deceased donors

Five-hundred and nine KTRs receiving kidneys from 386 DDs were included. Among the 386 DDs, 278 (72.0%) were classified as SCDs, and 108 (28.0%) were classified as ECDs. Ten DDs (5.2%) were diagnosed as AKI according to the decrease of urine output, and other 183 (94.8%) DDs were diagnosed according to the increase of serum creatinine level. In the SCD group, 112 (40.3%) donors had AKI; in the ECD group, 81 (75.0%) donors had AKI. KTRs were classified into the SCD-KT or ECD-KT group and each group was subdivided into an AKI donor group and a non-AKI donor group. Regarding distribution, 350 cases of SCD-KT group and 159 cases of ECD-KT group were analyzed (Fig. 1).

### Comparison of baseline characteristics between the SCD and ECD groups and between the SCD-KT and ECD-KT groups

The median follow-up period of this study was 48.1 (interquartile range 29.6–75.3) months. The mean age of the ECD group was higher than that of the SCD group (58.4 ± 5.2 vs. 38.9 ± 13.4 years, *p* < 0.001). The proportions of HTN, DM, and death due to cerebrovascular accident (CVA) were significantly higher in the ECD group than in the SCD group (47.6% vs. 13.4%, *p* < 0.001, 18.1% vs. 5.5%, *p* < 0.001 and 82.4% vs. 72.7%, *p* = 0.049, respectively). The baseline MDRD eGFR was significantly lower in the ECD group than in the SCD group (58.8 ± 23.6 ml/min/1.73 m^2^ vs. 76.0 ± 44.9 ml/min/1.73 m^2^, *p* < 0.001). The proportion of AKI was significantly higher in the ECD group than in the SCD group, but there was no significant difference according to the AKI stage between the two groups. All donors with AKI stage 3 took hemodialysis prior to organ procurement (Table 1).

**Table 1:** Comparison of clinical and laboratory parameters between expanded criteria donor (or recipient) and standard criteria donor (or recipient)

Variables	SCD (*n* = 278)	ECD (*n* = 108)	p for Trend
Donors			
Age at KT (years)	38.9 ± 13.4	58.4 ± 5.2	< 0.001
Gender (Male: Female)	188: 90	79: 29	0.327
BMI (kg/m^2^)	22.7 ± 3.8	23.2 ± 2.7	0.153
HTN, n (%)	36 (13.4)	50 (47.6)	< 0.001
DM, n (%)	15 (5.5)	19 (18.1)	< 0.001
Cause of donor death - CVA, n (%)	202 (72.7)	89 (82.4)	0.049
Baseline eGFR (ml/min/1.73 m^2^)	76.0 ± 44.9	58.8 ± 23.6	< 0.001
CKD stage 3 or above stage, n (%)	137 (49.5)	68 (63.0)	0.017
AKI, n (%)	112 (40.3)	81 (75.0)	< 0.001
Stage 1	43 (38.4)	22 (27.2)	
Stage 2	32 (28.6)	28 (34.6)	
Stage 3	37 (33.0)	31 (38.3)	
	SCD-KT (*n* = 350)	ECD-KT (*n* = 159)	p for Trend
Recipients			
Transplant centers, n (%)			0.083
A	256 (73.1)	122 (76.7)	
B	21 (6.0)	15 (9.4)	
C	73 (20.9)	22 (13.8)	
Transplant year, n (%)			0.005
1996–2005	35 (10.2)	6 (3.8)	
2006–2010	74 (21.1)	21 (13.2)	
2011–2016	241 (68.9)	132 (83.0)	
Age at KT (year)	46.6 ± 10.1	51.1 ± 9.4	< 0.001
Gender (Male: Female)	191: 159	100: 59	0.083
BMI (kg/m^2^)	22.5 ± 3.1	23.4 ± 3.4	0.003
HTN, n (%)	297 (84.9)	139 (87.4)	0.497
DM, n (%)	55 (15.7)	43 (27.0)	0.004
Dialysis before KT, n (%)	344 (98.3)	158 (99.4)	0.443
Dialysis duration, years	7.3 ± 4.6	7.6 ± 13.0	0.801
Previous KT, n (%)	41 (11.7)	10 (6.3)	0.079
Cause of ESRD, n (%)			0.001
Glomerulonephritis	165 (47.1)	53 (33.3)	
DM	45 (12.9)	36 (22.6)	
HTN	57 (16.3)	40 (25.2)	
Others	83 (23.7)	30 (18.9)	
Cold ischemic time (min)	244 ± 131	240 ± 110	0.749
HLA mismatch number	3.7 ± 1.4	3.7 ± 1.5	0.646
Induction, n (%)			0.070
Basiliximab	279 (79.7)	114 (71.7)	
ATG	70 (20.0)	45 (28.3)	
Main immunosuppressant, n (%)			
Tacrolimus: Cyclosporine	316: 33	153: 6	0.035
PRA > 50%	52 (18.6)	20 (13.7)	0.222

Values are expressed as means ± SDs, or n (%)

*SCD* standard criteria donor, *ECD* expanded criteria donor, *KT* kidney transplantation, *BMI* body mass index, *HTN* hypertension, *DM* diabetes mellitus, *CVA* cerebrovascular accident, *eGFR* estimated glomerular filtration rate, *CKD* chronic kidney disease, *AKI* acute kidney injury, *ESRD* end-stage renal disease, *HLA* human leukocyte antigen, *ATG* antithymocyte globulin, *PRA* panel reactive antibody

Regarding the recipients, the mean age and BMI were significantly higher in the ECD-KT group than in the SCD-KT group (51.1 ± 9.4 vs. 46.6 ± 10.1 years, *p* < 0.001, 23.4 ± 3.4 vs. 22.5 ± 3.1, *p* = 0.003, respectively). The proportion of KTRs with DM was significantly higher in the ECD-KT group than in the SCD-KT group (27.0% vs. 15.7%, *p* = 0.004) (Table 1). In the comparison of baseline characteristics between the AKI and non-AKI subgroups within the SCD-KT or ECD-KT group, donors were significantly younger in the AKI-ECD-KT subgroup than in the non-AKI-ECD-KT subgroup (*p* < 0.001) (Table 2).

**Table 2:** Comparison of clinical and laboratory parameters according to acute kidney injury in expanded criteria donor or standard criteria donor kidney transplantation

Variables	SCD-KT			ECD-KT		
	Non-AKI-KT	AKI-KT	p for Trend	Non-AKI-KT	AKI-KT	p for Trend
Donors	*n* = 166	*n* = 112		*n* = 27	*n* = 81	
Age at KT (years)	38.3 ± 14.5	39.9 ± 11.8	0.329	61.0 ± 3.4	57.5 ± 5.4	< 0.001
Gender (Male: Female)	107: 59	81: 31	0.192	16: 11	63: 18	0.079
BMI (kg/m^2^)	21.9 ± 3.7	23.8 ± 3.8	< 0.001	23.9 ± 2.7	23.0 ± 2.7	0.156
HTN, n (%)	23 (14.5)	13 (11.8)	0.588	15 (55.6)	35 (44.9)	0.377
DM, n (%)	11 (6.8)	4 (3.6)	0.293	3 (11.1)	16 (20.5)	0.388
Cause of donor death - CVA, n (%)	114 (68.7)	88 (78.6)	0.076	23 (85.2)	66 (81.5)	0.777
Recipients	*n* = 196	*n* = 154		*n* = 43	*n* = 116	
Age at KT (yr)	46.3 ± 9.5	47.1 ± 11.0	0.340	51.9 ± 9.3	50.8 ± 9.5	0.524
Gender (Male: Female)	105: 91	86: 68	0.746	29: 14	71: 45	0.580
BMI (kg/m^2^)	22.5 ± 3.3	22.5 ± 2.9	0.681	23.6 ± 3.2	23.3 ± 3.4	0.681
HTN, n (%)	163 (83.2)	134 (87.0)	0.369	36 (83.7)	103 (88.8)	0.423
DM, n (%)	26 (13.3)	29 (18.8)	0.183	11 (25.6)	32 (27.6)	0.844
Dialysis before KT, n (%)	193 (98.5)	151 (98.1)	1.000	42 (97.7)	116 (100.0)	0.270
Dialysis duration, years	7.8 ± 4.9	6.8 ± 4.3	0.321	5.9 ± 4.5	8.3 ± 15.0	0.305
Previous KT, n (%)	21 (10.7)	20 (13.0)	0.616	2 (4.7)	8 (6.9)	0.730
Cause of ESRD, n (%)			0.013			0.768
Glomerulonephritis	107 (54.6)	58 (37.7)		15 (34.9)	38 (32.8)	
DM	20 (10.2)	25 (16.2)		9 (20.9)	27 (23.3)	
HTN	30 (15.3)	27 (17.5)		9 (20.9)	31 (26.7)	
Others	39 (19.9)	44 (28.6)		10 (23.3)	20 (17.2)	
Cold ischemic time (min)	243 ± 132	245 ± 131	0.927	235 ± 99	242 ± 115	0.716
HLA mismatch number	3.6 ± 1.5	3.8 ± 1.2	0.241	3.6 ± 1.6	3.8 ± 1.4	0.442
Induction, n (%)			0.001			0.239
Basiliximab	169 (86.2)	111 (72.1)		34 (79.1)	80 (69.0)	
ATG	27 (13.8)	43 (27.9)		9 (20.9)	36 (31.0)	
Main immunosuppressant, n (%)						
Tacrolimus: Cyclosporine	175: 20	141: 13	0.769	40: 3	113: 3	0.345
PRA > 50%	30 (20.8)	22 (16.3)	0.359	4 (10.5)	16 (14.8)	0.595

Values are expressed as means ± SDs, n (%)

*SCD* standard criteria donor, *ECD* expanded criteria donor, *AKI* acute kidney injury, *KT* kidney transplantation, *BMI* body mass index, *HTN* hypertension, *DM* diabetes mellitus, *CVA* cerebrovascular accident, *ESRD* end-stage renal disease, *HLA* human leukocyte antigen, *ATG* antithymocyte globulin, *PRA* panel reactive antibody

### Comparison of the impact of donor AKI on the development of delayed graft function and changes in allograft function between the SCD-KT and ECD-KT groups

**Fig. 2:**
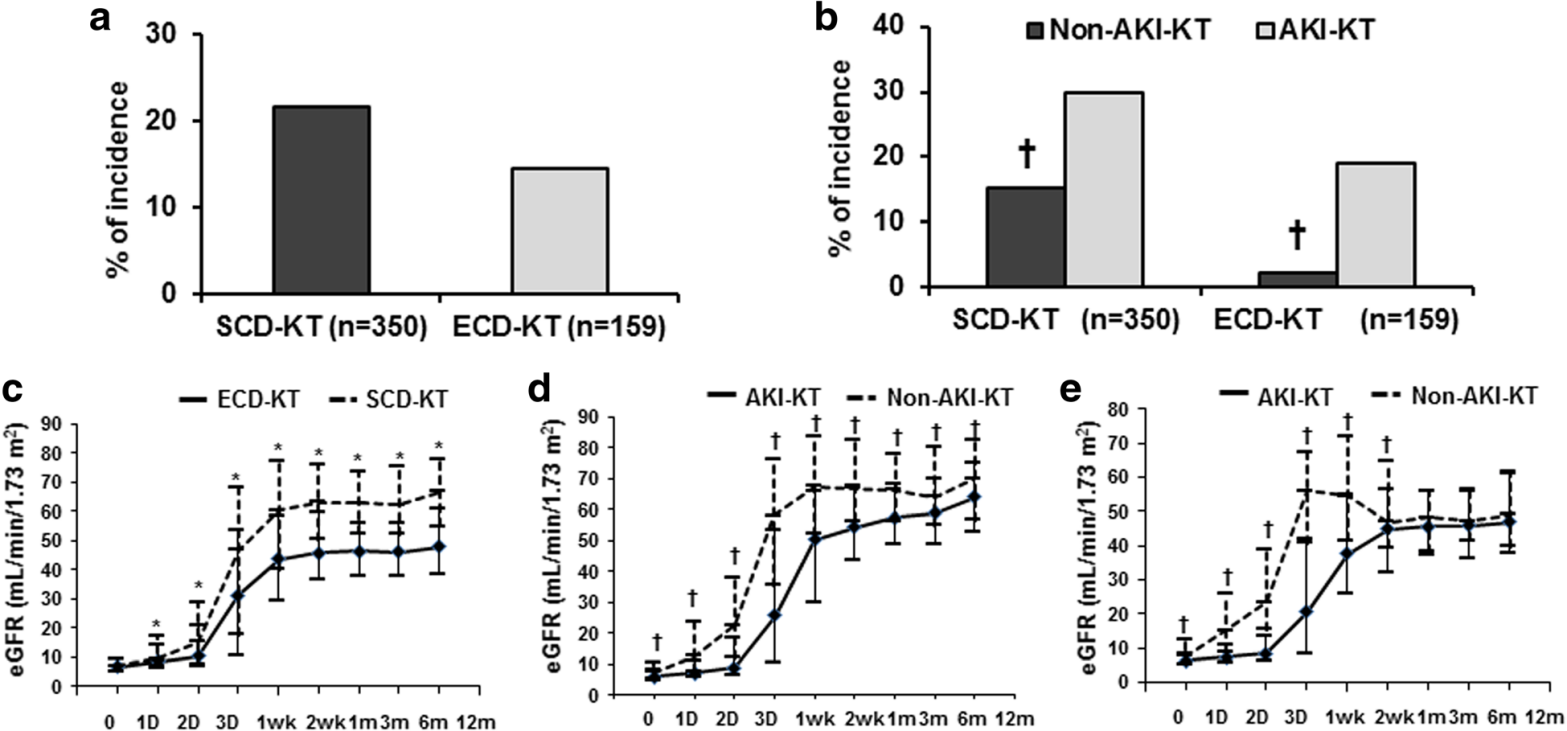
Comparison of the incidence of DGF (**a**); between the SCD-KT and ECD-KT groups and (**b**); between the non-AKI-KT and AKI-KT subgroups in the SCD-KT or ECD-KT group. The graph also shows a comparison of the change in allograft function after KT (**c**); between the SCD-KT and ECD-KT groups and between the non-AKI-KT and AKI-KT subgroups in (**d**); the SCD-KT group and (**e**); the ECD-KT group. *p < 0.05 vs. ECD-KT, †*p* < 0.05 vs. AKI-KT. Abbreviations: DGF, delayed graft function; SCD, standard criteria donor; KT, kidney transplantation; ECD, expanded criteria donor; AKI, acute kidney injury

The incidence of DGF was not significantly different between the SCD-KT and ECD-KT groups (Fig. 2a). In a subgroup analysis, the incidence of DGF was significantly higher in the AKI-KT subgroup than in the non-AKI-KT subgroup in both SCD-KT and ECD-KT groups (*p* = 0.005, *p* = 0.002, respectively) (Fig. 2b). In multivariate analysis, AKI was a significant risk factor for the development of DGF, but ECD was not. AKI and ECD did not show significant interaction effect (*p* = 0.998) (Table 3).

**Table 3:** Odd ratios for delayed graft function on the status of acute kidney injury or expanded criteria donor in deceased donor

	Unadjusted OR (95% C.I.)	p	Adjusted OR^a^ (95% C.I.)	p	*p*-value for interaction
AKI-KT	2.270 (1.423–3.621)	0.001	5.077 (2.564–10.055	< 0.001	0.998
ECD-KT	0.607 (0.365–1.011)	0.055	0.557 (0.293–1.056)	0.073	

*OR* odds ratio, *C.I*. confidence interval, *AKI* acute kidney injury, *KT* kidney transplantation, *ECD* expanded criteria donor

^a^Adjusted by recipient age, transplant year, transplant center, prior KT, recipient diabetes, HLA mismatch number, high PRA, donor gender

Allograft function through the first 12 months post-KT was significantly lower in the ECD-KT group compared with the SCD-KT group (*p* < 0.05) (Fig. 2c). In the subgroup analysis of the SCD-KT group, allograft function was significantly lower in the AKI-SCD-KT subgroup compared with the non-AKI-SCD-KT subgroup (*p* < 0.05) (Fig. 2d). In the ECD-KT group, allograft function was significantly lower in the AKI-ECD-KT subgroup until 3 months post-KT (*p* < 0.05); however, no difference was observed after this time (Fig. 2e).

### Comparison of the impact of donor AKI on the chronic allograft tissue injury between the SCD-KT and ECD-KT groups

We analyzed the findings of allograft biopsies obtained within 3 months from KT in 243 KTRs (Additional file 1: Figure S1). Regarding the allograft biopsies performed within 3 months, proportions of allograft tissue with IF (a) and TA (b) were significantly higher in the ECD-KT group than in the SCD-KT group (38.8% vs. 18.6%, *p* = 0.010 and 38.8% vs. 18.6%, *p* = 0.010, respectively). The mean GS percentage was significantly higher in the ECD-KT group than in the SCD-KT group (17.5 ± 23.3% vs. 5.0 ± 9.2%, *p* = 0.001) (c). In the SCD-KT group, there was no significant difference in the proportion of allograft tissue with IF, TA, or the mean GS percentage between the non-AKI-SCD-KT and AKI-SCD-KT subgroups. In contrast, in the ECD-KT group, the proportion of allograft tissue with IF, TA, and the mean GS percentage all tended to be higher in the AKI-ECD-KT subgroup than in the non-AKI-ECD-KT subgroup (d, e, f).

### Comparison of the impact of AKI in donors on the death-censored allograft survival between the SCD-KT and ECD-KT groups

**Fig. 3:**
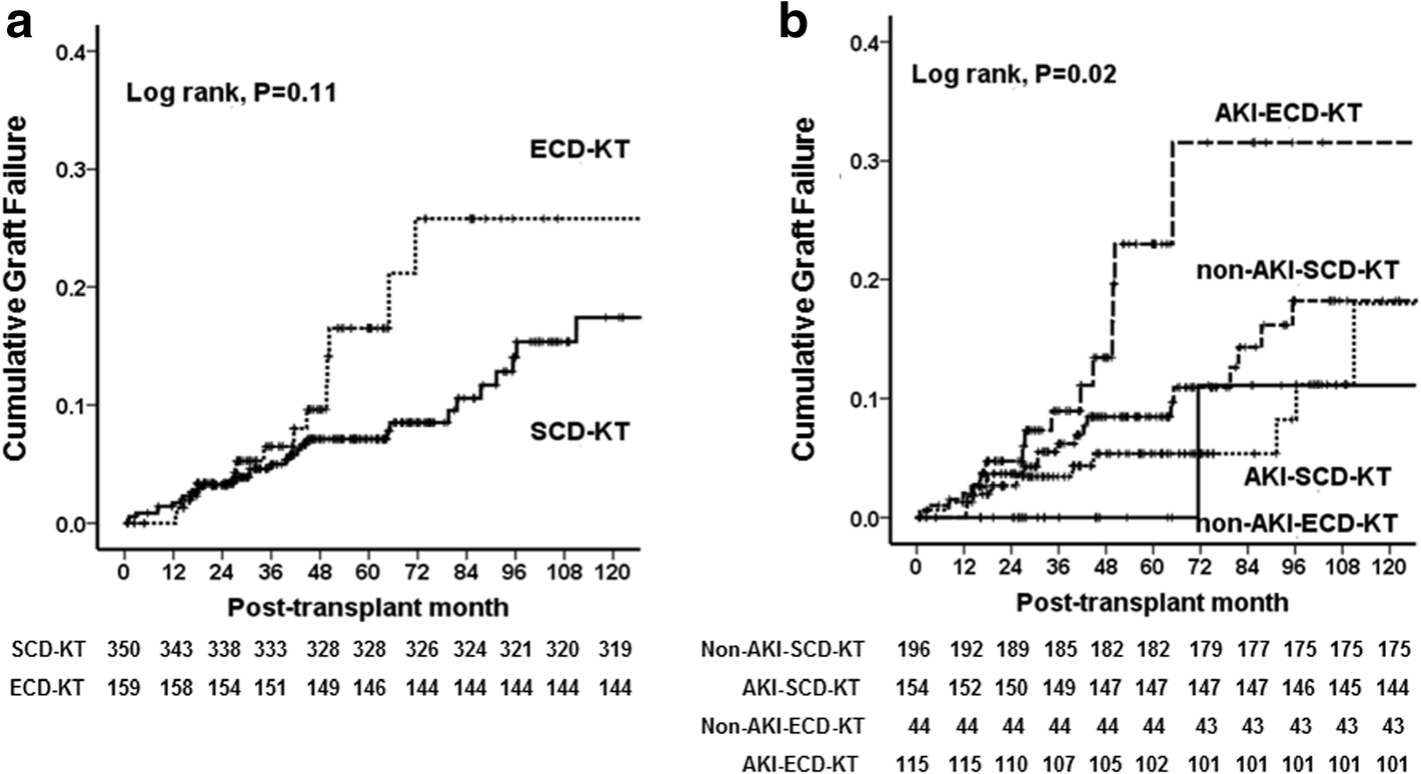
Comparison of the death-censored graft survival rate (**a**); between the SCD-KT and ECD-KT groups, and among (**b**); non-AKI-SCD-KT, AKI-SCD-KT, non-AKI-ECD-KT and AKI-ECD-KT, respectively. Abbreviations: SCD, standard criteria donor; KT, kidney transplantation; ECD, expanded criteria donor; AKI, acute kidney injury

A total of 73 cases (14.3%) of graft failure developed, including 52 (14.9%) cases in the SCD-KT group (33 patients in the non-AKI-SCD-KT subgroup and 19 patients in the AKI-SCD-KT subgroup) and 21 in the ECD-KT group (2 patients in the non-AKI-ECD-KT subgroup and 19 patients in the AKI-ECD-KT subgroup). No significant difference was detected in the causes of allograft failure between the SCD-KT and ECD-KT groups or between the AKI-KT and non-AKI-KT subgroups within the SCD-KT and ECD-KT groups (Table 4). Death-censored allograft survival tended to be higher in the SCD-KT group than in the ECD-KT group, but this difference was not significant (Fig. 3a). Meanwhile, it was significantly lower in the AKI-ECD subgroup in comparison with other 3 groups. However, AKI-SCD-KT or non-AKI-ECD-KT subgroup did not show a significant difference to non-AKI-SCD-KT group (Fig. 3b). Table 5 shows the incidence of allograft failure and the hazard ratios. The highest incidence of allograft failure was observed in AKI-ECD-KT subgroup. The predictors for allograft failure were evaluated using non-AKI-SCD-KT subgroup as the reference group. In multivariate analysis, KTRs from DDs with both AKI and ECD had the highest risks of the allograft failure after adjustment for recipient age, recipient diabetes, high PRA, transplant year, transplant center. There was a significant interaction between AKI in DDs and ECD for the allograft failure (*p* for interactio*n* = 0.007). However, a significant hazard ratio for allograft failure was not observed in KTRs from DD with either AKI or ECD only.

**Table 4:** Comparison of causes of graft failure and patient death according to acute kidney injury in expanded criteria donor or standard criteria donor kidney transplantation

Variables	SCD-KT			ECD-KT		
	Non-AKI-KT	AKI-KT	p for Trend	Non-AKI-KT	AKI-KT	p for Trend
Cause of graft failure, n (%)	*n* = 33	*n* = 19	0.290	*n* = 2	*n* = 19	0.429
Acute rejection	11 (33.3)	4 (21.1)		0	4 (21.1)	
Chronic rejection	5 (15.2)	5 (26.3)		1 (50.0)	7 (36.8)	
Recurrent glomerulonephritis	2 (6.1)	1 (5.3)		1 (50.0)	1 (5.3)	
BK virus-associated nephropathy	2 (6.1)	0		0	1 (5.3)	
Infection	2 (6.1)	0		0	1 (5.3)	
Ischemic injury	1 (3.0)	2 (10.5)		0	0	
Patient death with functioning graft	10 (30.0)	7 (36.8)		0	5 (26.3)	
Cause of patient death, n (%)	*n* = 13	*n* = 7	1.000	*n* = 0	*n* = 7	ns
Cardiovascular disease	4 (30.8)	2 (28.6)		0	3 (42.9)	
Infection	4 (30.8)	3 (42.9)		0	3 (42.9)	
Malignancy	3 (23.1)	1 (14.3)		0	0	
Bleeding	1 (7.7)	0		0	0	
Unknown	1 (7.7)	1 (14.3)		0	0	
Cerebrovascular accident	0	0		0	1 (14.3)	

Values are expressed as n (%)

*SCD* standard criteria donor, *KT* kidney transplantation, *ECD* expanded criteria donor, *AKI* acute kidney injury

**Table 5:** Incidence and hazard ratios of death-censored allograft failure on the status of acute kidney injury or expanded criteria donor in deceased donor

	No. of events	P	Unadjusted HR (95% C.I.)	p	Adjusted HR^a^ (95% C.I.)	p	*p*-value for interaction
Non-AKI-SCD-KT	24 (12.2%)	0.290	Reference		Reference		0.007
AKI-SCD-KT	12 (7.8%)		0.745 (0.419–1.323)	0.315	0.836 (0.403–1.734)	0.630	
Non-AKI-ECD-KT	2 (4.7%)		0.521 (0.180–1.523)	0.231	0.309 (0.041–2.351)	0.257	
AKI-ECD-KT	14 (12.1%)		2.175 (1.234–3.832)	0.007	2.122 (1.034–4.353)	0.040	

Values are expressed as n (%)

^a^Adjusted by recipient age, recipient diabetes, high PRA, transplant year, transplant center

*No* number, *HR* hazard ratio, *AKI* acute kidney injury, *SCD* standard criteria donor, *ECD* expanded criteria donosr

### Comparison of the impact of donor AKI on patient survival between the SCD-KT and ECD-KT groups

**Fig. 4:**
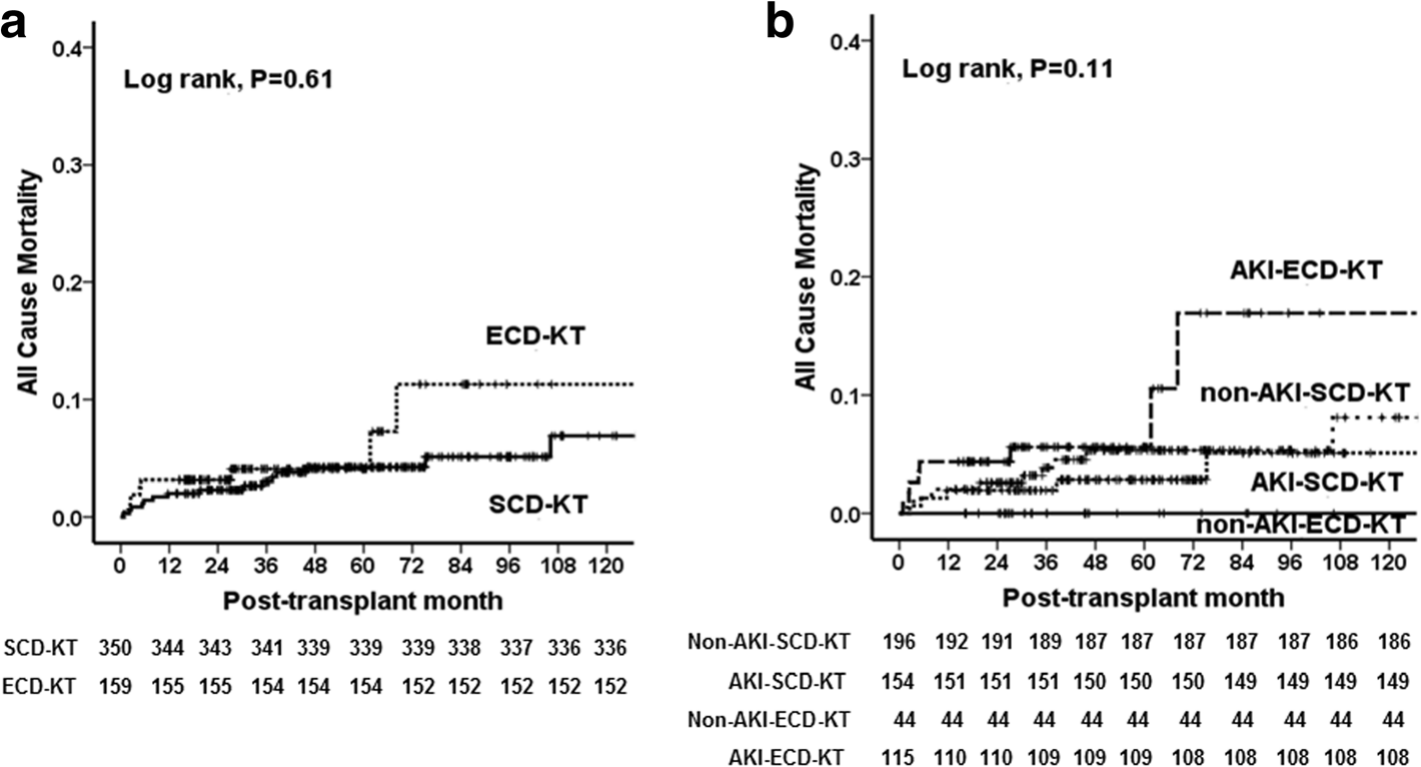
Comparison of patient survival rates (**a**); between the SCD-KT and ECD-KT groups, and among (**b**); non-AKI-SCD-KT, AKI-SCD-KT, non-AKI-ECD-KT and AKI-ECD-KT, respectively. Abbreviations: SCD, standard criteria donor; KT, kidney transplantation; ECD, expanded criteria donor; AKI, acute kidney injury

A total of 27 patients (5.3%) died, 20 of whom were in the SCD-KT group (13 patients in the non-AKI-SCD-KT subgroup and 7 patients in the AKI-SCD-KT subgroup) and 7 of whom were in the ECD-KT group (all 7 in the AKI-ECD-KT subgroup). The causes of death of the KTRs in the SCD-KT group were as follows: cardiovascular disease, 6 (30.0%); infection, 7 (35.0%); malignancy, 4 (20.0%); gastrointestinal bleeding 1, (5.0%); and unknown cause 2 (10.0%). In the ECD-KT group, the causes of death were as follows: cardiovascular disease, 3 (42.9%); infection, 3 (42.9%); and CVA, 1 (14.3%). There was no significant difference in the cause of patient death (Table 4) or in the patient survival rate between the SCD-KT and ECD-KT groups (*p* = 0.61) (Fig. 4a). When we compared patient survival among 4 groups (non-AKI-SCD-KT, AKI-SCD-KT, non-AKI-ECD-KT and AKI-ECD-KT), there was no significant difference (*p* = 0.11) (Fig. 4b).

## Discussion

In this study, we found that AKI considerably impacted post-transplant allograft survival when the DDs were classified as ECDs, whereas AKI did not have a significant impact when the DDs were SCDs. Our results suggest that strategies for preventing or minimizing the development of AKI in DDs, especially in ECDs, might help to improve allograft outcomes.

First, we compared the clinical characteristics of ECDs with those of SCDs. Donor age and the incidences of HTN, CVA, and AKI should be higher in ECDs because these factors define ECD [21]. Although DM was not included in the ECD criteria, the incidence was significantly higher in ECDs than in SCDs, perhaps because ECDs were significantly older than SCDs. Since the presence of DM or HTN can suggest underlying chronic tissue injury irrespective of allograft function, such donors could be diagnosed with CKD [26]. In addition, baseline allograft function as calculated by the MDRD equation was significantly lower in ECDs than in SCDs. Moreover, the proportion of donors with eGFR less than 60 mL/min/1.73 m^2^, which can be diagnosed as stage 3 or advanced stage CKD, was significantly higher in ECDs than in SCDs [26]. All of the above findings suggest that a significantly higher proportion of ECDs have underlying CKD compared with SCDs.

In comparison of the short-term clinical outcomes between the SCD-KT and ECD-KT groups, the incidence of DGF tended to be higher in the SCD-KT group than the ECD-KT group (*p* = 0.054), although this difference was not significant. In contrast, the incidence of DGF was higher in the AKI subgroup than in the non-AKI subgroup in both the SCD-KT and ECD-KT groups. Moreover, AKI was shown to be an independent risk factor for DGF in multivariate analysis, and that is consistent with previous studies [16, 18]. These findings suggest that recently developed AKI might have a more significant impact on the development of DGF compared to the baseline chronic damage of the allograft [10].

Meanwhile, allograft function at 6 months and 12 months post-transplant was lower in the ECD-KT group. The function might be lower because the baseline capacity to recover is lower in the ECD-KT group than in the SCD-KT group, as demonstrated by the lower baseline eGFR before KT (Table 1). Comparison of the AKI-KT and non-AKI-KT subgroups revealed that allograft function tended to deteriorate during the early post-transplant period. However, at about 1 year after KT, the allograft function of the two subgroups within the SCD-KT and ECD-KT were no longer significantly different, consistent with previous studies [10, 16, 27].

When we analyzed the chronic tissue injury score using allograft tissue obtained within 3 months of KT, the Banff scores (IF/TA, GS) associated with chronic damage were significantly higher in the ECD-KT group than in the SCD-KT group. Especially, they were significantly higher or showed higher tendency in the AKI-ECD-KT subgroup than other three subgroups, but it did not differ among those three subgroups. It is difficult to determine the reason AKI-ECD-KT subgroup had exclusively advanced chronic allograft tissue injury than other subgroups. However, as we described above, a significant proportion of ECDs might have underlying CKD. Moreover, the duration from KT to allograft biopsy (< 3 months) is relatively short for significant chronic tissue injury to develop in allograft kidneys after KT. Hence, this finding might represent the baseline status of the donated kidneys before KT. In addition, AKI can accelerate chronic kidney injury in CKD [19, 20, 28]. Previous study also suggests that allograft biopsy findings in the early period actually may reflect acute changes related to recovery from donor AKI [27]. Therefore, AKI in DDs may be associated with the higher IF/TA scores detected in post-transplant biopsy findings in the AKI-ECD-KT subgroup than other subgroups.

Our main interest is that whether AKI in DDs has different impact on the long-term allograft survival between the SCD-KT and ECD-KT groups. There was no significant difference between the SCD-KT and ECD-KT groups in death-censored allograft survival. In addition, there was no difference between the AKI-KT and non-AKI-KT subgroups, which is consistent with our previous reports (data not shown) [16, 17]. However, when we did subgroup analysis, AKI-ECD-KT subgroup showed significantly worse allograft survival than all other three subgroups (non-AKI-ECD-KT, non-AKI-SCD-KT and AKI-SCD-KT subgroup). In multivariate analysis using Cox regression hazard model, co-existence of AKI and ECD in donor was a significant contributor to allograft failure and we also found significant interaction between AKI in DDs and ECD on allograft failure. However, either AKI in DDs or ECD alone did not show significant impact. Above findings suggest that AKI in DDs may have a significant impact on allograft outcomes in ECD-KT, but not in SCD-KT group.

On the contrary, patient survival was not significantly different between the SCD-KT and ECD-KT groups, and the distribution of the cause of death did not depend on AKI. In the ECD-KT group, the AKI-ECD-KT subgroup tended to have worse outcomes, but these differences were not significant. It may be because patient death rate was too low to draw any conclusion about this issue. Another possible reason is that, because recipient age had such a strong effect on patient survival, the impact of other factors could appear as shown in the previous studies [29–32]. However, further investigation is required to clarify this issue.

There are some limitations to our study. First, it is a retrospective study; therefore, there is a possibility of selection bias. However, we note that our study has analyzed a large number of KTRs from multiple centers, and we have adjusted our result according to transplant centers and also transplant year in the multivariate analysis. Second, we could not analyze allograft biopsy findings from all patients because the biopsies were performed in only about half of the patients. In addition, we did not perform serial biopsies to understand the extent of the causal relationship between the more advanced chronic allograft tissue injury and higher rate of allograft failure in the AKI-ECD-KT subgroup than other three groups. Lastly, we used ECD criteria instead of more recently introduced Kidney Donor Profile Index (KDPI). However, the usefulness of KDPI has not been fully investigated, hence we decided to use ECD criteria in this study.

## Conclusion

In conclusion, the results of this study suggest that AKI superimposed on ECDs has a synergistically adverse impact on the long-term post-transplant allograft outcomes in the corresponding recipients. Therefore, careful monitoring and strategies for protecting against AKI may be required especially in ECD, to prevent its adverse effect on the post-transplant allograft outcomes.

## Additional file

Additional file 1: **Figure S1.** Comparison of the proportion of allograft tissue with chronic tissue injury: (a) IF, (b) TA, and (c) mean GS percentage between the SCD-KT and ECD-KT groups or (d) IF, (e) TA, and (f) mean GS percentage between the non-AKI-KT and AKI-KT subgroups within the SCD-KT or ECD-KT group. Data were obtained from allograft tissue obtained within 3 months after KT. *p < 0.05 vs. ECD-KT, †*p* < 0.05 vs. AKI-KT. Abbreviations: IF, interstitial fibrosis; TA, tubular atrophy; GS; glomerulosclerosis; SCD, standard criteria donor; KT, kidney transplantation; ECD, expanded criteria donor; AKI, acute kidney injury. (TIF 1061 kb)

## Abbreviations

AKI: Acute kidney injury
BMI: Body mass index
CKD: Chronic kidney disease
CVA: Cerebrovascular accident
DDKT: Deceased donor kidney transplantation
DDs: Deceased donors
DGF: Delayed graft function
DM: Diabetes mellitus
ECDs: Expanded criteria donors
eGFR: Estimated glomerular filtration rate
GS: Glomerulosclerosis
HLA: Human leukocyte antigen
HTN: Hypertension
IF: Interstitial fibrosis
KDIGO: Kidney Disease: Improving Global Outcomes
KT: Kidney transplantation
KTRs: Kidney transplant recipients
MDRD: Modification of diet in renal disease
PRAs: Panel reactive antibodies
SCDs: Standard criteria donors
SD: Standard deviation
TA: Tubular atrophy
UNOS: United Network for Organ Sharing

## References

[1] Goldberg D, Kallan MJ, Fu L, Ciccarone M, Ramirez J, Rosenberg P, Arnold J, Segal G, Moritsugu KP, Nathan H (2017). Changing metrics of organ procurement organization performance in order to increase organ donation rates in the United States. Am J Transplant.

[2] Girlanda R (2016). Deceased organ donation for transplantation: challenges and opportunities. World J Transplant.

[3] Saidi RF, Hejazii Kenari SK (2014). Challenges of organ shortage for transplantation: solutions and opportunities. Int J Organ Transplant Med.

[4] Keith DS, Vranic GM (2016). Approach to the highly sensitized kidney transplant candidate. Clin J Am Soc Nephrol.

[5] Park WY, Kang SS, Park SB, Park UJ, Kim HT, Cho WH, Han S (2016). Comparison of clinical outcomes between ABO-compatible and ABO-incompatible spousal donor kidney transplantation. Kidney Res Clin Pract..

[6] Snanoudj R, Timsit MO, Rabant M, Tinel C, Lazareth H, Lamhaut L, Martinez F, Legendre C (2017). Dual kidney transplantation: is it worth it?. Transplantation.

[7] Perez-Saez MJ, Montero N, Redondo-Pachon D, Crespo M, Pascual J (2017). Strategies for an expanded use of kidneys from elderly donors. Transplantation.

[8] Ojo AO (2005). Expanded criteria donors: process and outcomes. Semin Dial.

[9] Savoye E, Tamarelle D, Chalem Y, Rebibou JM, Tuppin P (2007). Survival benefits of kidney transplantation with expanded criteria deceased donors in patients aged 60 years and over. Transplantation.

[10] Klein R, Galante NZ, de Sandes-Freitas TV, de Franco MF, Tedesco-Silva H, Medina-Pestana JO (2013). Transplantation with kidneys retrieved from deceased donors with acute renal failure. Transplantation.

[11] Farney AC, Rogers J, Orlando G, al-Geizawi S, Buckley M, Farooq U, al-Shraideh Y, Stratta RJ (2013). Evolving experience using kidneys from deceased donors with terminal acute kidney injury. J Am Coll Surg.

[12] van Ittersum FJ, Hemke AC, Dekker FW, Hilbrands LB, Christiaans MH, Roodnat JI, Hoitsma AJ, van Diepen M (2017). Increased risk of graft failure and mortality in Dutch recipients receiving an expanded criteria donor kidney transplant. Transpl Int.

[13] Querard AH, Foucher Y, Combescure C, Dantan E, Larmet D, Lorent M, Pouteau LM, Giral M, Gillaizeau F (2016). Comparison of survival outcomes between expanded criteria donor and standard criteria donor kidney transplant recipients: a systematic review and meta-analysis. Transpl Int.

[14] Wu VC, Wu PC, Wu CH, Huang TM, Chang CH, Tsai PR, Ko WJ, Chen L, Wang CY, Chu TS (2014). The impact of acute kidney injury on the long-term risk of stroke. J Am Heart Assoc.

[15] Davenport A (2008). The brain and the kidney--organ cross talk and interactions. Blood Purif.

[16] Lee MH, Jeong EG, Chang JY, Kim Y, Kim JI, Moon IS, Choi BS, Park CW, Yang CW, Kim YS (2014). Clinical outcome of kidney transplantation from deceased donors with acute kidney injury by acute kidney injury network criteria. J Crit Care.

[17] Kim JH, Kim YS, Choi MS, Kim YO, Yoon SA, Kim JI, Moon IS, Choi BS, Park CW, Yang CW (2017). Prediction of clinical outcomes after kidney transplantation from deceased donors with acute kidney injury: a comparison of the KDIGO and AKIN criteria. BMC Nephrol.

[18] Boffa C, van de Leemkolk F, Curnow E, Homan van der Heide J, Gilbert J, Sharples E, Ploeg RJ (2017). Transplantation of kidneys from donors with acute kidney injury: friend or foe?. Am J Transplant.

[19] Ishani A, Xue JL, Himmelfarb J, Eggers PW, Kimmel PL, Molitoris BA, Collins AJ (2009). Acute kidney injury increases risk of ESRD among elderly. J Am Soc Nephrol.

[20] Hsu CY, Ordonez JD, Chertow GM, Fan D, McCulloch CE, Go AS (2008). The risk of acute renal failure in patients with chronic kidney disease. Kidney Int.

[21] Metzger RA, Delmonico FL, Feng S, Port FK, Wynn JJ, Merion RM (2003). Expanded criteria donors for kidney transplantation. Am J Transplant.

[22] Khwaja A (2012). KDIGO clinical practice guidelines for acute kidney injury. Nephron Clin Pract.

[23] Sis B, Mengel M, Haas M, Colvin RB, Halloran PF, Racusen LC, Solez K, Baldwin WM, Bracamonte ER, Broecker V (2010). Banff '09 meeting report: antibody mediated graft deterioration and implementation of Banff working groups. Am J Transplant.

[24] Yarlagadda SG, Coca SG, Garg AX, Doshi M, Poggio E, Marcus RJ, Parikh CR (2008). Marked variation in the definition and diagnosis of delayed graft function: a systematic review. Nephrol Dial Transplant.

[25] Levey AS, Coresh J, Greene T, Marsh J, Stevens LA, Kusek JW, Van Lente F (2007). Chronic kidney disease epidemiology collaboration. Expressing the modification of diet in renal disease study equation for estimating glomerular filtration rate with standardized serum creatinine values. Clin Chem.

[26] Levey AS, Eckardt KU, Tsukamoto Y, Levin A, Coresh J, Rossert J, De Zeeuw D, Hostetter TH, Lameire N, Eknoyan G (2005). Definition and classification of chronic kidney disease: a position statement from kidney disease: improving global outcomes (KDIGO). Kidney Int.

[27] Heilman RL, Smith ML, Kurian SM, Huskey J, Batra RK, Chakkera HA, Katariya NN, Khamash H, Moss A, Salomon DR (2015). Transplanting kidneys from deceased donors with severe acute kidney injury. Am J Transplant.

[28] Lim SY, Lee JY, Yang JH, Na YJ, Kim MG, Jo SK, Cho WY (2016). Predictive factors of acute kidney injury in patients undergoing rectal surgery. Kidney Res Clin Pract.

[29] Foucher Y, Akl A, Rousseau V, Trebern-Launay K, Lorent M, Kessler M, Ladriere M, Legendre C, Kreis H, Rostaing L (2014). An alternative approach to estimate age-related mortality of kidney transplant recipients compared to the general population: results in favor of old-to-old transplantations. Transpl Int.

[30] Hernandez D, Rufino M, Bartolomei S, Lorenzo V, Gonzalez-Rinne A, Torres A (2005). A novel prognostic index for mortality in renal transplant recipients after hospitalization. Transplantation.

[31] Hernandez D, Sanchez-Fructuoso A, Gonzalez-Posada JM, Arias M, Campistol JM, Rufino M, Morales JM, Moreso F, Perez G, Torres A (2009). A novel risk score for mortality in renal transplant recipients beyond the first posttransplant year. Transplantation.

[32] Jassal SV, Schaubel DE, Fenton SS (2005). Predicting mortality after kidney transplantation: a clinical tool. Transpl Int.

