# Supplementary figures and images for: Impact of acute kidney injury in expanded criteria deceased donors on post-transplant clinical outcomes: multicenter cohort study
Source: BMC Nephrol. 2019 Feb 4;20:39. doi: 10.1186/s12882-019-1225-1 (PMC6360778; doi:10.1186/s12882-019-1225-1)

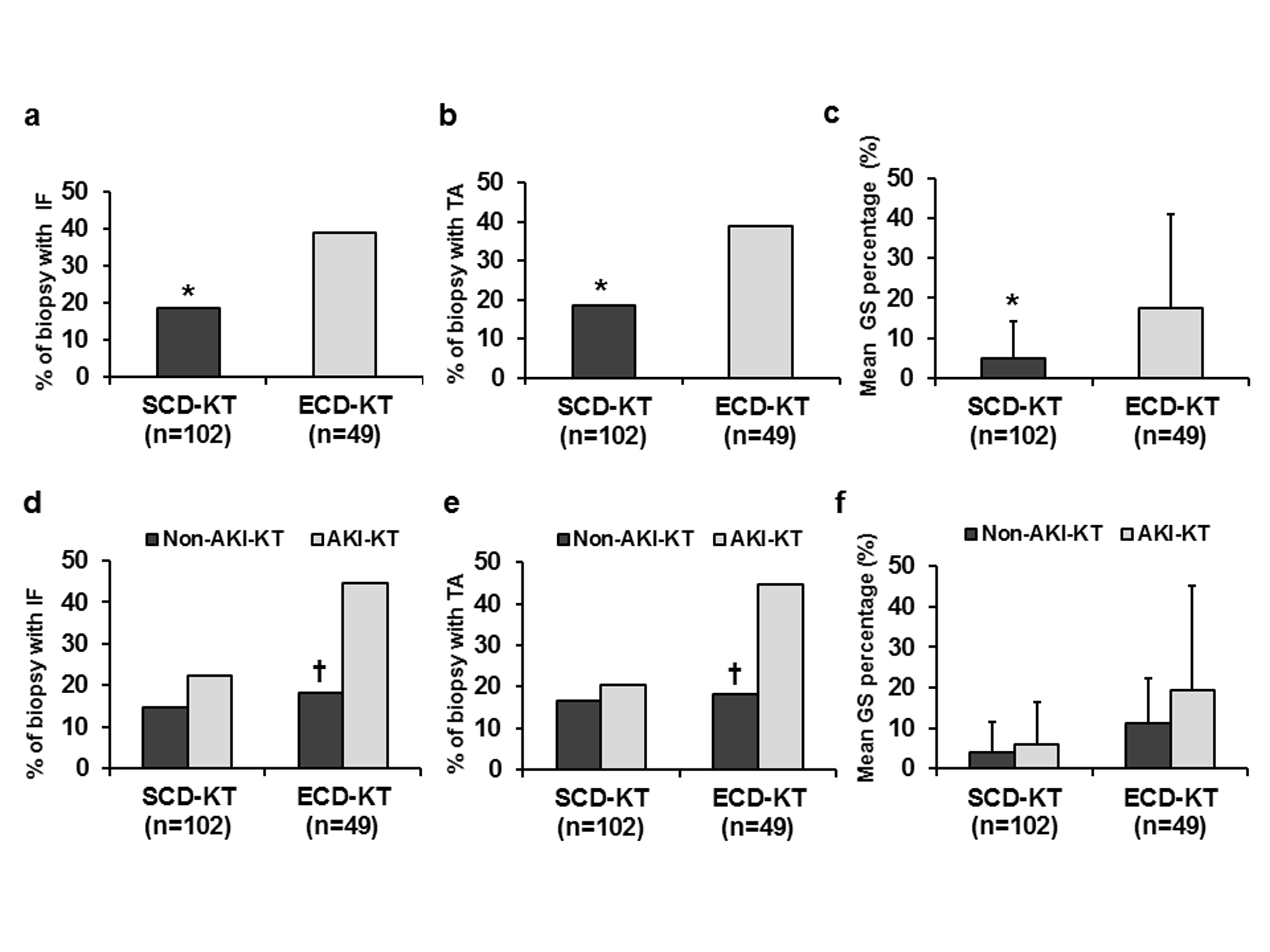

Supplement: Supplementary file 1 — Figure S1. Comparison of the proportion of allograft tissue with chronic tissue injury: (a) IF, (b) TA, and (c) mean GS percentage between the SCD-KT and ECD-KT groups or (d) IF, (e) TA, and (f) mean GS percentage between the non-AKI-KT and AKI-KT subgroups within the SCD-KT or ECD-KT group. Data were obtained from allograft tissue obtained within 3 months after KT. *p < 0.05 vs. ECD-KT, †p < 0.05 vs. AKI-KT. Abbreviations: IF, interstitial fibrosis; TA, tubular atrophy; GS; glomerulosclerosis; SCD, standard criteria donor; KT, kidney transplantation; ECD, expanded criteria donor; AKI, acute kidney injury. (TIF 1061 kb) [file 12882_2019_1225_MOESM1_ESM.tif]
